# IMPlementing IMProved Asthma self-management as RouTine (IMP^2^ART) in primary care: study protocol for a cluster randomised controlled implementation trial

**DOI:** 10.1186/s13063-023-07253-9

**Published:** 2023-04-03

**Authors:** Kirstie McClatchey, Vicky Hammersley, Liz Steed, Jessica Sheringham, Viv Marsh, Atena Barat, Brigitte Delaney, Thomas Hamborg, Deborah Fitzsimmons, Steve Holmes, Tracy Jackson, Elisabeth Ehrlich, Noelle Morgan, Ann Saxon, Megan Preston, David Price, Stephanie J. C. Taylor, Hilary Pinnock, the IMP2 ART Programme Group

**Affiliations:** 1 Asthma UK Centre for Applied Research, Old Medical School, Usher Institute, The University of Edinburgh, Teviot Place, Edinburgh, EH8 9AG UK; 2 Wolfson Institute of Population Health, Mary University of London, London, Queen UK; 3 Department of Applied Health Research, University College London, London, UK; 4 School of Health and Related Research, The University of Sheffield, Sheffield, UK; 5 Faculty of Medicine, Health and Life Science, Swansea Centre for Health Economics, Swansea University, Swansea, UK; 6 The Park Medical Practice, Shepton Mallet, UK; 7 Severn School of Primary Care, Health Education England (South West), Bristol, UK; 8 Education for Health, Warwick, UK; 9 Optimum Patient Care, Cambridge, UK; 10 Observational and Pragmatic Research Institute, Singapore, Singapore; 11 Centre of Academic Primary Care, Division of Applied Health Sciences, University of Aberdeen, Aberdeen, UK

**Keywords:** Protocol, Randomised controlled implementation trial, IMP^2^ART, Asthma, Self-management, Primary care

## Abstract

**Background:**

Asthma is a common long-term condition and major public health problem. Supported self-management for asthma that includes a written personalised asthma action plan, supported by regular professional review, reduces unscheduled consultations and improves asthma outcomes and quality of life. However, despite unequivocal inter/national guideline recommendations, supported self-management is poorly implemented in practice. The IMPlementing IMProved Asthma self-management as RouTine (IMP^2^ART) implementation strategy has been developed to address this challenge. The aim of this implementation trial is to determine whether facilitated delivery of the IMP^2^ART strategy increases the provision of asthma action plans and reduces unscheduled care in the context of routine UK primary care.

**Methods:**

IMP^2^ART is a parallel group, cluster randomised controlled hybrid II implementation trial. One hundred forty-four general practices will be randomly assigned to either the IMP^2^ART implementation strategy or control group. Following a facilitation workshop, implementation group practices will receive organisational resources to help them prioritise supported self-management (including audit and feedback; an IMP^2^ART asthma review template), training for professionals and resources to support patients to self-manage their asthma. The control group will continue with usual asthma care. The primary clinical outcome is the between-group difference in unscheduled care in the second year after randomisation (i.e. between 12 and 24 months post-randomisation) assessed from routine data. Additionally, a primary implementation outcome of asthma action plan ownership at 12 months will be assessed by questionnaire to a random sub-group of people with asthma. Secondary outcomes include the number of asthma reviews conducted, prescribing outcomes (reliever medication and oral steroids), asthma symptom control, patients’ confidence in self-management and professional support and resource use. A health economic analysis will assess cost-effectiveness, and a mixed methods process evaluation will explore implementation, fidelity and adaptation.

**Discussion:**

The evidence for supported asthma self-management is overwhelming. This study will add to the literature regarding strategies that can effectively implement supported self-management in primary care to reduce unscheduled consultations and improve asthma outcomes and quality of life.

**Trial registration:**

ISRCTN15448074. Registered on 2 December 2019.

**Supplementary Information:**

The online version contains supplementary material available at 10.1186/s13063-023-07253-9.

## Background

An estimated 3.6 million people in the United Kingdom (UK) are actively being treated for asthma [1]. Each year, asthma is responsible for over 6 million primary care consultations, nearly 100,000 hospital admissions [1] and over 1000 deaths (20 a year in children under 14 years)[2], at a cost to the NHS in England and Wales of at least £1billion [1]. Societal costs accumulate throughout life with asthma-related absence from school or work, disability and premature retirement. Much of this morbidity is preventable with appropriate/timely (self) management [3–5].

Our systematic meta-review, funded by the National Institute for Health and Care Research (NIHR) Health Service and Delivery Research (HS&DR), synthesised evidence from 27 systematic reviews (270 RCTs) and concluded that supported self-management reduces hospitalisations, accident and emergency (A&E) attendances and unscheduled consultations, and improves markers of control and quality-of-life for people with asthma [6]. A written personalised asthma action plan, completed as part of a self-management discussion and reviewed regularly, empowers patients to recognise deterioration and take appropriate action (e.g. increasing/commencing medication; seeking medical help)[7–9]. The cost of providing self-management support (estimated in a recent network meta-analysis for asthma as a 2-h investment in the first year [10]) is offset by the reduction in hospitalisations and unscheduled healthcare [6]. Effectiveness of supported self-management has been demonstrated in diverse cultural groups [11–14], children [15–17], adolescents [18, 19], adults [7] and elderly populations [20, 21], and in both primary/secondary healthcare settings [22–25]. A range of modes of delivery (including telehealth) [25–29] may be used to suit preferences and context.

For three decades [30], national and international guidelines have recommended—unequivocally—that people with asthma should be provided with self-management education, reinforced by a personalised action plan and supported by regular review with a healthcare professional [3–5]. Implementation, however, remains poor in routine clinical practice. Surveys from the UK, USA, Northern Europe and Australia reveal that less than a third of people with asthma have an action plan [31–34]. Routine primary care data from our developmental work revealed that only 6% had a record of being given an action plan [35]. In 2014, the UK National Review of Asthma Deaths highlighted that half the people who died had not accessed medical help, emphasising the vital importance of asthma self-management to facilitate timely response to deteriorating asthma control [36].

The solution will need a whole system approach [37]. An NIHR HS&DR-funded systematic review of the implementation of supported self-management concluded that whilst patient education, professional training and organisational support were all essential, they were rarely effective in isolation [38]. Effective implementation was multifaceted and multidisciplinary, engaging patients, training and motivating professionals, within the context of an organisation that actively supports self-management [39]. A systematic review of asthma implementation studies [39] identified small randomised controlled trials (RCTs) evaluating either patient education, professional training or organisational support and observational studies reporting whole system initiatives—including some very large effective national projects [40, 41]. There were no randomised trials evaluating whole system implementation strategies: a gap that the current study aims to address.

### Aims and objectives

IMP^2^ART (IMPlementing IMProved Asthma self-management as RouTine) is a UK-wide cluster randomised implementation trial that aims to test the impact of a whole system implementation strategy that embeds supported asthma self-management in primary care, compared with usual care on:

- Primary clinical outcome: unscheduled care
- Implementation outcome: ownership of an action plan
- Secondary outcomes (Number of asthma reviews conducted, prescribing of reliever medication and oral steroids, asthma symptom control, patients’ confidence in self-management and professional support)

A health economic evaluation will assess the costs from the perspective of the healthcare service and also from a societal perspective. A process evaluation will use mixed methods to explore feasibility/acceptability of the IMP^2^ART implementation strategy and explore how supported self-management was implemented (or not) by primary care practices to aid interpretation and inform scaling up and sustainability.

## Methods

### Study design and settings

The trial uses a parallel group, hybrid II (addressing both implementation and clinical outcomes) [42] cluster randomised controlled implementation trial design (randomisation at the general practice level), testing the implementation of an evidence-based and guideline-recommended intervention. The trial will be conducted in general practices across England and Scotland. The protocol adopts the principles and terminology of the Standards for Reporting Implementation studies (StaRI) [43], uses the Template for Intervention Description and Replication (TIDieR) guide [44] to describe the implementation strategy and follows the SPIRIT checklist [45] (Additional file 1) to report the trial methodology. In addition, the CONSERVE-SPIRIT checklist [46] guides reporting of modifications due to the COVID-19 pandemic.

### Practice eligibility criteria and recruitment

**Fig. 1:**
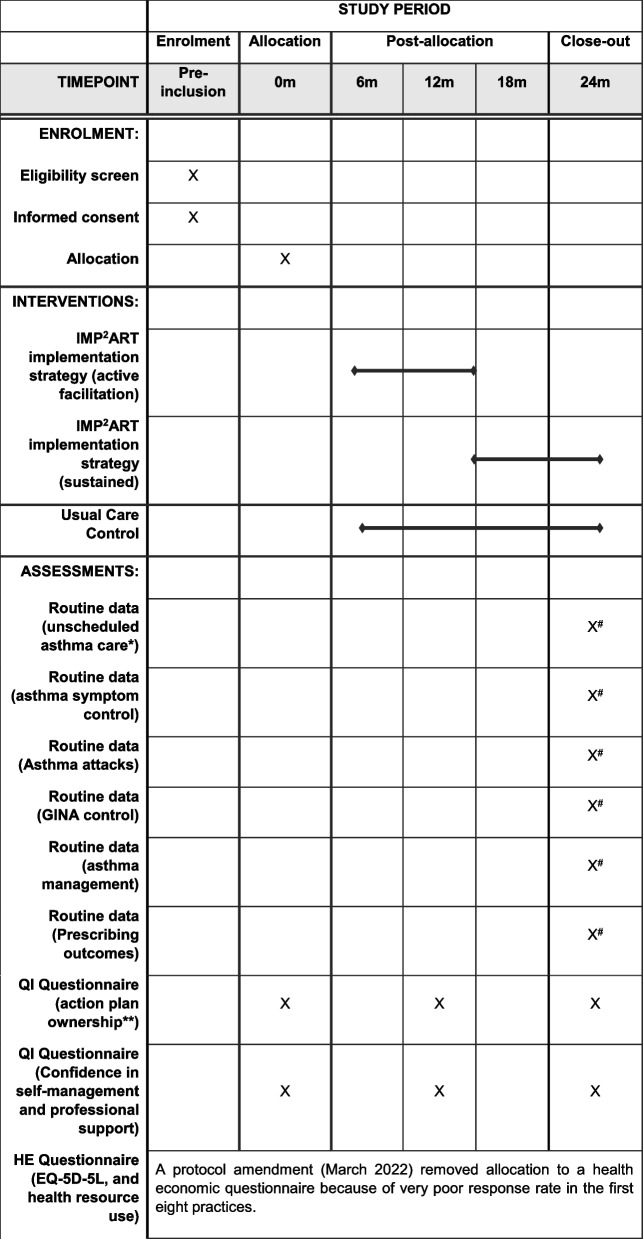
SPIRIT figure of enrollment, interventions and assessments

We will recruit 144 general practices in the UK (England and Scotland) and randomly assign them with a 1:1 ratio to the implementation or control arm. Figure 1 shows the SPIRIT figure of the stages of enrolment, intervention and outcome assessment.

To be eligible, practices must use one of the four common electronic health record (EHR) systems (EMIS, SystmOne, Vision or Microtest) for which components of our implementation strategy have been designed, and agree to Optimum Patient Care (OPC: a social enterprise that leads quality improvement initiatives involving routine data extraction from practices https://optimumpatientcare.org) extracting anonymised routine coded data to measure the primary and other outcomes of the study. A successful extraction of data is a requirement to (a) demonstrate that there are no insurmountable governance or technical problems, (b) establish that the practice data would allow assessment of the primary health outcome, and (c) for use in the baseline audit and feedback reports.

Practices will be of varying sizes (to reflect the range of UK primary care) and thus have different numbers of ‘active asthma’ patients. Active asthma is defined by the UK Quality and Outcome Framework (an annual reward and incentive programme for all general practices in England) as having a coded diagnosis of asthma and having been prescribed an asthma medication within the previous year [47]. Our sample size calculation takes variable cluster size into consideration, but we will exclude very small practices likely to have substantially fewer than 200 patients with ‘active asthma’ registered throughout the trial. (Note: this will be an estimate at the time of recruitment as we will not know the exact cluster sizes until the final routine data are available at the end of the trial). Finally, we will exclude practices undertaking research or initiatives that might affect our outcomes, and practices that work closely with another participating practice (e.g. as part of a network or federation). Decisions about potential contamination will be overseen by a sub-committee (chaired by SJCT).

### Clinically eligible patient population

This is a practice-level intervention and we are not recruiting individual patients to the trial. Our patient population is all people who are eligible clinically to be offered supported asthma self-management by the practice. In line with national/international guideline recommendations, this is all patients with a diagnosis of ‘active’ asthma [3–5, 47]. The only exclusions (which we will apply in defining our patient populations from routine data) are:

- Under 5 years of age. Standard approaches to asthma self-management are ineffective in this age group [3].
- Under the care of a severe/difficult asthma clinic [3–5], though supporting their specialist action plan is appropriate.
- Significant co-morbid chronic obstructive pulmonary disease [48], as this requires a COPD action plan.
- Identified by the practice as being clinically unsuitable (e.g. severe cognitive impairment, on the palliative care register (a list held by the practice of those under their care who may be approaching end of life)).

Our primary health outcome (unscheduled care) will be assessed on routine data from the whole eligible population as defined above (excluding any patient whose EHR is coded as not wanting their data used for any purpose other than their care) and who have been on the ‘active asthma’ register of the practice throughout the 3-year data collection period (1-year pre-trial and 2-years during the trial).

### The IMP^2^ART implementation strategy

**Fig. 2:**
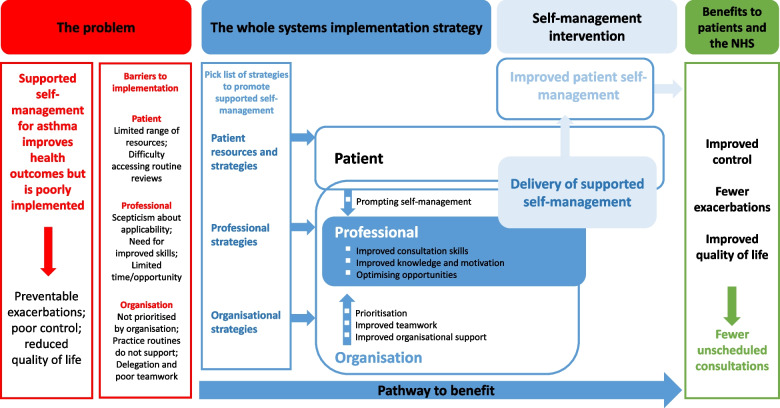
The IMP^2^ART logic model illustrating the pathway-to-benefit

Informed by our understanding of the literature, and building on our developmental work [49–51], we have illustrated our ‘pathway-to-benefit’ (Fig. 2), which displays the implementation strategy. General practices randomised to the implementation group will receive the whole system implementation strategy directed at patients, professionals and the organisation, and this will be supported by expert nurse facilitation for 12 months. Table 1 displays the implementation strategy.

**Table 1:** Components of the whole system IMP^2^ART implementation strategy, and adaptation for the COVID-19 pandemic

Target group	Strategy (bold is core content)	Description	COVID-19 modifications
Patient*	Asthma review invitation letters and SMS messages	Template asthma review invitation letters (including letters for annual reviews, missed review appointments, reviews following unscheduled care) that highlight the importance of reviews and asthma action plan ownership	Invitation letters adapted to include remote options, e.g. telephone/video-call
	Patient content of the *Living with Asthma* website	A range of online resources for people living with asthma (e.g. action plans, information about triggers) have been collated on patient-facing pages of the *Living with Asthma* website	Informed by a review of existing information about COVID-19 [52], tailored information about COVID-19 for those living with asthma and remote asthma review information added
	Waiting room posters	Waiting room posters for practices highlighting the importance of self-management and asthma action plans, and encouraging patients to speak to staff about these	No change
Professional**	**Module 1: Team module** [53]	A short introductory online module for the whole-practice team (e.g. administrative staff, nurses, GPs) to raise awareness of supported asthma self-management and the importance of team working. This interactive module will be introduced in the 1-h IMP^2^ART workshop, to facilitate discussion about IMP^2^ART strategies that the practice could adopt to support/embed self-management. Goals are set and summarised as the practice ‘team plan’	No change to the module, though the IMP^2^ART workshop was shifted to online
	**Module 2: Individual study **[53]	An in-depth online module for the individual(s) in the practice most involved with delivering asthma care. The module is designed for 60 min of independent study and aims to enable healthcare professionals to support effective self-management with confidence, and be motivated to adopt the IMP^2^ART resources identified in their practice’team plan’	Content was added to the module that covered effective remote consultation skills [53]
	Professional content of the *Living with Asthma* website	A range of online resources for healthcare professionals (e.g. action plans, patient information for use in reviews) have been collated on the professional pages of the *Living with Asthma* website	Added information and resources related to providing remote asthma reviews [54, 55]
Organisation***	Asthma Review Template [56, 57]	An asthma review template that will be embedded in practice systems (EMIS, SystmOne, Vision, Microtest) for use in asthma reviews. Templates will be ‘QOF-compliant’ [47], patient-centred, highlight action plan provision, and link to the *Living with Asthma* website	No change
	**Audit and feedback **[58]	Annual audit reports focusing on supported self-management will be provided at baseline, 12 and 24 months. Brief monthly reports will be delivered to practices for the duration of their participation in the trial, focusing on a summary of unscheduled care, patients reviewed and action plan provision compared to an OPC database average. The email containing the monthly report will include a ‘top tip’. Reports allow data to be de-anonymised by the practices, so that ‘at-risk’ patients can be identified and invited for review	Annual audit and feedback report updated to include a section on COVID-19: number/% of patients considered at high risk of complications due to COVID-19; number/% of patients with confirmed or suspected COVID-19; patient COVID-19 vaccination status (number/%)

^*^Developed with Patient and Public Involvement (PPI) colleagues

^**^Developed with Education for Health (EfH)

^***^Developed with Optimum Patient Care (OPC)

### Facilitation

Nurse specialists with facilitation experience will be trained to facilitate the implementation of IMP^2^ART within practices. The aim is to mimic the support that a Healthcare Trust might provide when promoting a new initiative. All implementation practices will receive an initial IMP^2^ART workshop either face-to-face or via Microsoft Teams (an approach that was added in response to COVID-19), at which the facilitator will consider the baseline audit with the practice team and introduce components of Module 1. The facilitator will guide the practice to develop their ‘team plan’ for implementing IMP^2^ART, discuss how core strategies (see Table 1) can be adopted/adapted to suit the practice routines, and identify additional strategies that might help individual practices. Facilitators will observe progress (by monitoring monthly audit reports), and offer additional support to practices struggling to implement supported self-management. Practices will receive the initial facilitated workshop and up to 10 h contact time (maximum 12 h) according to need over 12 months. A final contact at 12 months post-randomisation concludes the facilitated delivery of the implementation strategy, though all strategies (Table 1) remain available.

### Fidelity and adaptation

The core strategies will be delivered to all implementation practices including facilitation, access to Module 1, completion of Module 2 by at least one member of the clinical staff, and receipt of the audit and feedback reports. All implementation practices will be encouraged to arrange a date for the IMP^2^ART whole team workshop. If, however, a practice has not had a workshop 20 weeks post-randomisation, access to all strategies will be provided and a facilitator will offer informal support to ensure the practice is aware of all the resources. The workshop is intended to be delivered to the practice team, but if only a few members of a practice team are able to attend, arrangements for ‘cascade training’ will be discussed. Other strategies will be strongly promoted to all practices (use of the asthma review template and the patient resources), but adoption of these is optional, and practices will be encouraged to adapt the strategies to their practice routines (e.g. adding additional fields to the template to suit existing practice routines).

### Usual care in control group practices

General practices in the control arm of the trial will continue with their usual asthma care and will not receive any of the components of the implementation strategy. However, they will receive standard versions of the annual audit and feedback reports. This is an extensive report covering all aspects of asthma care that OPC routinely provide to practices contracted to their quality improvement service. In contrast to the IMP^2^ART implementation arm annual reports, there is no focus on supported self-management, and the feedback on action plan provision is towards the end of the report.

### Clinical care in both groups

Clinical care will be provided by the patients’ usual clinical advisors in accordance with the UK and/or global asthma guidelines [3–5] and according to the clinical needs of the patient.

### Adaptation of the implementation strategy in response to the COVID-19 pandemic

Although a number of practices were preparing to participate, none had been randomised when the trial was suspended in March 2020 (the start of the COVID-19 pandemic). The implementation strategy was thus not delivered pre-COVID. Between April and December 2020, we reviewed the whole IMP^2^ART implementation strategy with the help and advice of the IMP^2^ART Professional Advisory and Patient and Public Involvement Groups. The overarching strategies did not change, but we reviewed and (where necessary) adapted components to ensure they reflected the new context and the new routines in primary care (Table 1).

### Outcomes

#### Primary clinical outcome: unscheduled asthma care (second year post-randomisation)

The primary clinical outcome is the proportion of clinically eligible patients with at least one episode of unscheduled care for asthma in the second year after randomisation (supported self-management will be targeted by the implementation strategy during the first year post-randomisation, thus, impact on unscheduled care will not be apparent until the second year). Unscheduled care is defined as the proportion of people (≥ 5 years of age) with at least one unscheduled asthma consultation (GP consultation; and/or out-of-hours attendance; A&E attendance; hospital admission) during the second year in the trial (i.e. between 12 and 24 months post-randomisation). The primary clinical outcome will be measured using routine coded data extracted by OPC at 24 months from clinically eligible patients on the ‘active asthma’ register of participating practices. As there is considerable annual turnover in the ‘active asthma’ register (20% per year in one practice [59]), we will only include patients who have been on the ‘active asthma’ register of the practice at baseline, 12 months and 24 months.

#### Primary implementation outcome: ownership of action plans at 12 months

The primary implementation outcome is the proportion of patients with an action plan. We will measure action plan ownership by a quality improvement questionnaire mailed to 50 patients randomly selected from the clinically eligible population in 32 randomly selected practices (16 practices in each trial arm). Ownership of an action plan is defined as the proportion of people (≥ 5 years of age) who respond ‘Yes’ to the question ‘Has your asthma nurse or doctor provided you with an asthma “action plan”?’ in the OPC quality improvement questionnaire mailed at 12 months. The questionnaire will be mailed, with one reminder sent by SMS.

#### Secondary outcomes

Table 2 displays the secondary outcomes that will be assessed from routine data at baseline, 12 months and 24 months extracted by OPC under their service-level agreement with practices.

**Table 2:** Secondary outcomes

Secondary outcomes	Description
Asthma symptom control	We will assess asthma control using coded data for the Royal College of Physicians 3 Questions (RCP3Qs) [60]. Status with regard to these three questions is routinely recorded (in England) for the QoF. The clinical implications of the responses have been established [61]
Asthma attacks	We will report: • Unscheduled care in the first year post-randomisation as a secondary outcome (unscheduled care in the second year post-randomisation is the primary outcome) • The proportion of people prescribed a course of oral steroids in the previous 12 months (a recognised marker of a severe attack [62]), and the number of steroid courses/patient/year
GINA control	We will assess the composite outcome of ‘GINA control’. The GINA guidelines define control over a period of 4 weeks as no night-time symptoms or activity limitation, symptoms/requirement for rescue medication < 2 doses/week and no attacks in previous year [4]. We will assess this: • For the whole population from routine data (e.g. from responses to the RCP 3Qs, prescribing data), • For the randomly selected sub-group from the responses in the quality improvement questionnaire that enable assessment of GINA control
Prescribing outcomes	We will measure: • The proportion of people prescribed inhaled steroids and number of prescriptions/year; • The proportion of people prescribed reliever medication and number of prescriptions/year; • The proportion of people using a sub-optimal treatment regimen (defined as a ratio of the number of prescriptions for controller medication to total number of prescriptions for all asthma medication < 0.5 [63]
Asthma management	• Asthma reviews will be measured using the proportion of people with active asthma reviewed each year • Provision of action plans (as opposed to ownership which will be assessed in the quality improvement questionnaire) will be measured by assessing the proportion of people (≥ 5 years) who have a record of the provision/updating of an action plan in the previous 3 years assessed at 12 and 24 months post-randomisation
Confidence in self-management and professional support	The Asthma Bother Profile (management section) reflects quality of asthma care and patient’s confidence in ability to self-manage on a scale of 0 (no confidence) to 5 [64]. These questions will be included in the quality improvement questionnaire

### Sample size calculations

Sample size for primary clinical outcome of unscheduled care within the previous year.

In our preliminary work, we found an unscheduled care rate of 34% [35]. We chose an absolute difference of 7% (from 34 to 27%) between trial arms as being the minimum important effect clinically and to health services. This is approximately half the effect achieved in effectiveness trials of supported self-management [6]. To detect this difference with 90% power and 5% significance level, 1868 patients would be required in an individually randomised trial without clustering.

Assuming an intraclass correlation (ICC) of 0.07 (taken from experience of pragmatic implementation studies, and supported by our analysis of data from 379 OPC practices) and assuming 200 individuals/practice, we need 70 practices (14,000 patients) in each group (increased to 72 practices/arm to allow for practice withdrawal) to detect the minimum important effect with 90% power and 5% significance level. Patients will only be included in the analysis dataset if they have ‘active asthma’ with the same practice at baseline, 12 months and 24 months follow-up, which avoids individual patient attrition. We originally intended to limit recruitment to practices with a list size > 6000 (assuming 6% will have active asthma [65]) to avoid cluster sizes < 200 (and randomly sampling if clusters were > 200). During recruitment, it became clear that this excluded many small rural general practices in Scotland, so we opted to allow variable cluster sizes (including a few clusters likely to be < 200). Furthermore, the COVID-19 pandemic resulted in a reduction in asthma attacks [66, 67], and an analysis of the OPC dataset (*n* = 286 practices) in September 2021 showed that 25.8% was a more realistic estimate of the proportion with unscheduled care that we could expect in the control group. Maintaining the recruitment target of 144 practices (140 after loss to follow-up) and allowing for a variable cluster size (mean = 200; coefficient of cluster size variation = 0.8), the study would have 94.7% power to detect a reduction from 25.8 to 18.8%.

### Sample size for the implementation outcome of asthma plan ownership

About a third of people who responded to an Asthma and Lung UK survey in 2014 owned an action plan [68]. Assuming a 15% increase in asthma action plan ownership in the IMP^2^ART implementation arm, we would have an effect size of *h* = 0.322. To detect this difference with 90% power, 203 patients in each arm would be needed without clustering. Accounting for within general practice clustering with ICC = 0.03, we would need 20 completed questionnaires from 32 clusters (16 practices from each arm), a total of *n* = 640 patients. Given the average questionnaire response rate is ≈45%, we will post questionnaires to 50 participants/practice.

### Randomisation

The unit of randomisation will be the general practice. Remote online randomisation (1:1 implementation:control) will be provided by the Pragmatic Clinical Trials Unit (PCTU), Queen Mary University of London. Stratifiers, determined according to contextual influences identified during developmental work [50, 51], will be deprivation status (less deprived, ≤ median deprivation score/more deprived, > median deprivation score), practice list size (small, ≤ 8035 patients; large, > 8035 patients) and GP training status of the practice (yes/no). In a second step, 32 practices will be randomly selected for quality improvement questionnaire data collection, ensuring that these practices are distributed evenly between the implementation and control groups. Randomisation is implemented in REDCap (Research Electronic Data Capture) software [69] and allocations will be requested by the programme manager (VH).

### Protection against bias

Blinding of general practices to allocation will not be possible; however, aspects of the trial data collection and analysis will be blind. Routine data collection for the primary health outcome (unscheduled care) will be collected by automated software used by OPC for their quality improvement service. The personnel who produce audit reports for the practices (who cannot be blinded) will not be the same as the OPC research database personnel who will handle data for the trial and who will be blind to allocation. OPC’s quality improvement questionnaires (including the primary implementation outcome: ownership of action plans) are self-completed. Data entry and data cleaning personnel will be blind to allocation. Statistical analysis will be undertaken by personnel blind to allocation.

### Statistical analysis

Analysis will be on data at 12 months (implementation/process outcomes) and 24 months (health outcomes) follow-up. Individuals will be included in the analysis only if they were registered in a practice at baseline, 12 months and 24 months follow-up. Practices will be analysed in the group to which they were allocated regardless of compliance (intention-to-treat). The target of estimation is the average treatment effect across participants in terms of the primary clinical as well as primary implementation outcome. An unweighted independence estimating equation analysis on participant-level data will be used to estimate the participant-average difference in proportions between groups. This model employs an independence working correlation structure in conjunction with robust standard errors to account for clustering. Secondary outcomes will be analysed using the same or a similar model appropriate for the respective variable type. The models will include fixed effects for stratification variables and may incorporate additional individual-level covariates as fixed effects (covariates are limited as we are taking them from routine data). A fully pre-specified model will be provided in the statistical analysis plan (SAP). Treatment effect estimates, with associated confidence intervals and *p*-values, and ICCs will be reported for each outcome.

The primary clinical outcome as well as the secondary outcomes of unscheduled care (12 months), asthma attacks and prescribing outcomes will, by definition, not have any missing data as the absence of a code describing an event or prescription is defined as the binary variable value ‘no’. Patterns and amount of missing data for questionnaire outcomes and other routine data variables will be explored, and a suitable multiple imputation method used. The strategy for dealing with missing values will be articulated in detail in the SAP which will be prepared by the Pragmatic Clinical Trials Unit (PCTU) statisticians in consultation with other blinded research team members. Allocation codes will not be released to the statisticians before the SAP is signed off and made publicly available.

### Health economic evaluation

A health economic analysis from the perspective of the UK NHS and Personal Social Services will be conducted alongside the trial. A detailed health economic analysis plan (HEAP) will be produced alongside the SAP, including strategies for dealing with missing data. We will use routine data at 24 months to capture a detailed profile of healthcare resource use (e.g. unscheduled care, prescriptions) in both implementation and control practices. Resources associated with implementing the IMP^2^ART strategy will be determined from trial data. Resource use will be valued in £ sterling using published unit costs [70].

Using the primary outcome, a cost-effectiveness analysis will be undertaken with appropriate, discounting of costs and benefits (3.5%). We will undertake deterministic sensitivity analyses to assess the impact of parameter changes on our base-case incremental cost-effectiveness ratio (ICER). In order to provide a comprehensive picture of the health economic findings, costs will be tabulated for all other outcomes, as part of a cost-consequences approach [71].

We will undertake a cost-utility analysis to estimate the incremental cost per quality-adjusted life year (QALY) of IMP^2^ART compared to control at 24 months. A decision analytical model will be constructed, informed by structured literature searches and in consultation with the IMP^2^ART team. We will derive health utilities from the literature. All model inputs and assumptions will be agreed with the IMP^2^ART experts for the base-case analysis and inform the parameters and assumptions to be tested in sensitivity/scenario analysis.

A cost-utility analysis will examine the incremental cost/QALY. QALY gains or losses will also be used in a net–benefit analysis based on accepted NICE ‘value for money’ thresholds. Deterministic sensitivity analysis will assess the impact of parameter variation on the baseline estimates. Probabilistic sensitivity analysis will investigate the joint uncertainty in parameter values, and cost-effectiveness acceptability curves will illustrate uncertainty surrounding the estimates of cost-effectiveness and probability (in % likelihood) of the intervention being cost-effective against society’s willingness to pay, using NICE thresholds [72]. We will explore longer-term cost-effectiveness, using estimates derived from the trial and information from literature sources relating to longer-term costs and effects (QALYs) to arrive at plausible long-term estimates of cost-effectiveness.

### Process evaluation

The trial will include a nested process evaluation to understand the national/local practice context and changes over time, assess fidelity and/or adaptation of the implementation strategy, assess receipt and response to the implementation strategy (i.e. how much of the different components was actually received and taken up by practice staff), explore reach and potential for scaling-up and explore sustainability to inform if/how the intervention is embedded. Using established case study methodology [73], we will conduct in-depth case studies in up to four implementation practices to understand their process of implementing IMP^2^ART. The case studies will involve: interviews (*n*≈12/case study) with individuals who deliver supported self-management in the practice; observation of activities (*n*≈10–20 h/case study), e.g. training sessions, practice meetings; documentary analysis (»40/case), e.g. anonymised notes and plans, meeting minutes; audio-recording asthma clinics (*n* = 3/case study) to assess delivery of supported self-management within routine reviews.

Additionally, facilitator log books will be completed throughout the trial, and an exit survey for all practices will be conducted. Up to 12 interviews with key informants (e.g. practice nurses, GPs, practice managers) will be undertaken in non-case study practices to understand specific aspects of implementation of supported self-management in practices, responses to IMP^2^ART strategies, and to explore the transferability of preliminary case study findings. Additional focussed interviews (*n*≈10) will be conducted with national/regional opinion leaders, healthcare managers and policymakers, to explore policy perspectives and/or generalisability of emerging themes. We will interview all facilitators (*n*≈4) about their experience of facilitating the delivery of IMP^2^ART.

Implementation fidelity with be assessed and will include both adoption and adaptation of the implementation strategy. We will follow the National Institutes of Health Behaviour Change Consortium’s fidelity framework [74] to consider the five aspects of fidelity (study design, training providers, treatment delivery, treatment receipt, treatment enactment). This will include the following: assessment of facilitator training and facilitator delivery of IMP^2^ART (video-recording of the IMP^2^ART workshop), treatment receipt will be monitored by uptake of skills by practices and use of implementation strategies. We will follow FRAME-IS to report adaptations to the implementation strategy [75].

A mixed methods analysis will be conducted for the process evaluation to understand the extent to which delivery or response to IMP^2^ART strategies or the national/local context influenced the outcomes achieved. Qualitative and quantitative analysis will be undertaken concurrently with findings from each used to inform the interpretation of the other.

### Impact of the COVID-19 pandemic on outcomes

In addition to affecting trial timelines and requiring intervention modifications the pandemic also had an impact on trial outcomes and consequently the statistical analysis. Restrictions on movement and interaction with people outside of one’s household were imposed leading to reduced viral spread and reduced access to healthcare (the public were encouraged to access healthcare only when absolutely necessary) [66, 67]. There is also evidence [66] of increased demand for preventer medication at the onset of the lockdown (March 2020) though whether this represents improved adherence to medication or stockpiling is unclear. Our analysis of a large dataset provided by OPC research database confirmed marked changes in primary and secondary outcomes in the first year of the pandemic compared to the prior year.

The original intention was that statistical analysis models would adjust for baseline, that is, the 12 months prior to randomisation. The main analysis of outcomes has now been changed to models not adjusting for baseline as the effect of the pandemic on baseline outcomes will differ for clusters. For practices recruited earlier, the baseline period will overlap with the pandemic and associated restrictions to a large extent whilst for practices recruited later there were fewer, if any, restrictions (note that there were also regional variations in restrictions). Consideration was given to using the 12-month period prior to the pandemic as the baseline. This was also rejected as (a) the time period between baseline and follow-up would vary for clusters and (b) for participants to be included in the study data they would have to be a registered patient with that practice at all three time points, and this longer overall time period would have reduced the sample size. However, data will be extracted from the 12 months before the start of the pandemic until the 24 months follow-up ends and three sensitivity analyses will be conducted—a model adjusting for baseline at the individual level, a model adjusting for baseline at the individual level using the 12 months before the pandemic as the baseline period, and a repeated measures model using baseline, 12-months and 24-month follow-up.

In addition, the pandemic also led to more GP consultations being conducted remotely (telephone or video). New codes for this were introduced which may have resulted in changes to routine data coding practices. Therefore, the code lists defining the primary clinical outcome and secondary outcome which were derived in an early phase of the programme needed to be updated.

### Stakeholder engagement

In addition to the multidisciplinary research team (academics, general practitioners (GPs), nurses, health psychologists, health economists), a Professional Advisory Group (led by SH) from members of the Primary Care Respiratory Society will meet regularly to offer advice on aspects of the IMP^2^ART implementation strategy, to contribute to raising the profile of the work, and to advise on longer-term sustainability. A Patient Advisory Group (led by TJ & NM) from the Asthma UK Centre Patient and Public Involvement (PPI) platform will be consulted regularly for their opinions on the development of the implementation components, research materials, interpretation of findings and dissemination.

### Internal pilot

The trial included an internal pilot completed between January 2021 and September 2021. The initial 12 practices were recruited as pilot practices with the aim of:

- Observing feasibility of the trial procedures (practice recruitment, initial data extraction, randomisation, mailing of the OPC quality improvement questionnaire, setting up the implementation strategy according to allocation).
- Exploring engagement with the IMP^2^ART implementation strategy (access to education modules, use of the patient-facing website, acceptability to the practice teams) and fidelity with which the components of the implementation strategy were adopted/adapted (video-recording of the facilitated IMP^2^ART workshops).

The trial processes for the 12 pilot practices proceeded as described for the main cluster-RCT (the remaining 132 practices), with the 12 practices randomised to either the implementation strategy or control. Process data from researcher and facilitator logs, and qualitative data from interviews with pilot practice staff were analysed to provide evidence for the funder’s progression criteria and inform the processes of the main trial. A number of minor adjustments were made to processes but no substantial changes were made to the implementation strategy or trial procedures [76].

### Ethical and governance considerations

The study has received ethical approval from NHS Lothian (REC No: 19/EM/0279), NRS and HRA approval (NRS Ref NRS/19/256672), and local governance as required in all areas. The decision of a practice to participate in the study is voluntary and will be based on a clear understanding of what is involved, detailed in an Organisational Information Document (OID) (Additional file 2). Practices will have sufficient time to consider the oral and written information provided, and to clarify points they do not understand. A practice may withdraw their consent to participate at any time.

The trial is overseen by an Independent Steering Committee, but a Data Monitoring and Ethics Committee is not appropriate as no outcome data will be available for monitoring until the end of the trial. No interim analysis is possible. The trial sponsor is the Academic and Clinical Central Office for Research and Development (ACCORD no: AC19081) who will assess risk and determine if an audit is required.

### Dissemination

At the end of the IMP^2^ART programme, we will write summaries providing feedback to the practices, including a lay summary suitable for sharing on the practice website.

At the end of the trial, we will hold workshops in each of the three research sites (London/Sheffield/Edinburgh) to which we will invite key stakeholders and participating practices. The aim will be to share preliminary findings and invite discussion on the interpretation and implications, to gauge applicability of our findings to a broader range of practices and contexts [77], and to maximise the study’s reach by inviting a broad range of stakeholders (patient groups, healthcare professionals, managers and commissioners, academics) not involved in IMP^2^ART. In addition, we will disseminate findings to healthcare practitioners, healthcare planners and policy makers, and also professional audiences, grant holders. Collaborators will use their professional networks to raise awareness of the programme of work.

Conference abstracts and peer-reviewed publications will report findings throughout the programme grant, with key publications in high-impact journals. We have adopted the terminology and will follow the StaRI reporting standards for implementation studies [43].

The authorship policy follows the ICMJE criteria for academic publications (http://www.icmje.org). If the journal allows, all publications resulting from IMP^2^ART programme will include in the authorship list ‘the IMP^2^ART Group’. Links to publications will be sent to practices that participated in the trial. Finally, we will use the innovative dissemination channels of the Asthma UK Centre for Applied Research (AUKCAR) [https://www.ed.ac.uk/usher/Aukcar] (websites/blogs/twitter/public lectures) to raise awareness of our publications. Asthma + Lung UK will support dissemination and promotion of our findings to their patient and scientific audiences using their digital and social media platforms.

## Discussion

This trial will test whether a whole system implementation programme (IMP^2^ART) can improve supported self-management for asthma in routine primary care practice in England and Scotland, as measured by a reduction in unscheduled care and an increase the provision of asthma action plans provided.

### Strengths and limitations

A major strength of the study is the robust randomised controlled design. Additionally, the trial and the implementation components have been designed by a multidisciplinary team, including engagement with PPI colleagues and professional stakeholders currently working in primary care, strengthening the applicability to real-world practice. The extensive developmental phases have ensured that the components of the implementation strategy are theoretically based and revised after feasibility testing [28, 49–58]. However, the trial has a number of limitations; for example, late-2020 changes to the QOF to include a written personalised action plan [78] may influence English practices interest in supported self-management, though this will affect practices in both groups equally. Use of routine data allows assessment of the impact of implementing supported self-management in the population of all clinically eligible people with asthma, and our procedures for blinding of routine data extraction, handling and analysis reduce bias. Although we cannot eliminate coding discrepancies, our preliminary work has enabled us to describe the likely impact on our outcomes. Finally, although the implementation strategy was adapted for a COVID-19 context, it is uncertain what implications the pandemic may have had on the trial, although again, this will affect practices in both groups equally.

### Desired impact

Supported self-management has been recommend by asthma guidelines for three decades, but the challenge of implementation means that only a minority of people with asthma benefit from the improved outcomes that it offers. IMP^2^ART explicitly addresses this challenge in the context of routine UK primary care and will inform future implementation and potentially improve the lives of people living with asthma.

### Trial status

*Protocol version:* 4.0, 19/04/22.

*Recruitment:* General practice recruitment began in January 2021, and we anticipate finalised randomisation at end of March 2023.

*Trial status:* Ongoing.

*Sponsor*: ACCORD, Royal Infirmary of Edinburgh, 47 Little France Cres, Edinburgh EH16 4TJ.

## Supplementary Information

**Additional file 1.**

**Additional file 2.**

## Data Availability

Due to the confidentiality of NHS routine data required for this trial, data will not be made available.

## Abbreviations

A&E: Accident and emergency
COVID-19: Coronavirus disease 2019
EHR: Electronic health record
GP: General Practitioner
HS&DR: Health Service and Delivery Research
ICC: Intraclass correlation
IMP^2^ART: IMPlementing IMProved Asthma self-management as RouTine
NIHR: National Institute for Health Research
OID: Organisational Information Document
OPC: Optimum Patient Care
PCTU: Pragmatic Clinical Trials Unit
PPI: Patient and Public Involvement
QALYs: Quality-adjusted life years
QOF: Quality and Outcomes Framework
RCP3Qs: Royal College of Physicians 3 Questions
RCT: Randomised controlled trial
REC: Research Ethics Committee
UK: United Kingdom
