# Supplementary material for: IMPlementing IMProved Asthma self-management as RouTine (IMP2ART) in primary care: study protocol for a cluster randomised controlled implementation trial
Source: Trials. 2023 Apr 3;24:252. doi: 10.1186/s13063-023-07253-9 (PMC10068707; doi:10.1186/s13063-023-07253-9)
Supplement: Supplementary file 2 — Additional file 2. [file 13063_2023_7253_MOESM2_ESM.docx]

# Organisation Information Document – Non-Commercially Sponsored Studies

**(Template version: 1.3)**

## Guidance on Using This Document

Please use this document to create the outline Organisation Information Document/s that you will submit with your IRAS Form. In most instances the Organisation Information Document should be localised before sharing with participating NHS / HSC organisations.

Questions/items marked with an asterisk* (Questions 1-3, 5, 8 and 12-15, as well as items throughout the appendices as applicable) must be completed prior to submission of the IRAS Form in all cases. Only if the localised Organisation Information Document is to be used as the Agreement between the parties should the Sponsor or authorised delegate check the relevant check-boxes at the top of each subsequent appendix and complete the authorisation section.

Items marked with a caret **^** are completed by the participating NHS / HSC organisation, after the Local Information Pack is shared and where relevant.

Remaining questions may be answered on the localised Organisation Information Document either by the Sponsor or authorised delegate prior to sharing the Local Information Pack, or by the participating NHS / HSC organisation (or collaboratively between the two) after the Local Information Pack is shared, as appropriate.

To provide an answer in the document, click in a box with the grey text (click here to enter text), choose the relevant option if presented with a drop-down list or click in the box if presented with a check-box ☐.

A separate guidance document is provided and should be consulted prior to completion of this document. Please also read the question specific guidance where present.

We welcome your feedback on the use of the UK Local Information Pack. If you would like to provide feedback, please take the [UK Local Information Pack Survey](https://wh.snapsurveys.com/s.asp?k=155862505933).

## Study Information

| 1.* IRAS Project ID | 256672 | |
| --- | --- | --- |
| **2.* Full Title of the Study** | Implementing supported asthma self-management in routine clinical care: designing, refining, piloting and evaluating clinical and cost-effectiveness of a whole systems implementation strategy (IMP2ART): Cluster Randomised Trial | |
| **3.* Legal Name(s) of Sponsor/Co-Sponsors/Joint-Sponsors** | University of Edinburgh/NHS Lothian | |
| 4. Contact details of person acting on behalf of Sponsor for questions relating to study set up. Please enter details of the person who is the Sponsor’s main point of contact for all correspondence on setting up the study at this NHS / HSC organisation. This contact may be the Sponsor, a Study Manager, Clinical Research Scientist or Study Coordinator. Where a Contract Research Organisation (CRO) or Clinical Trials Unit (CTU) has been delegated to handle set up on behalf of the Sponsor, the contact at the CRO or CTU should be named here. | | |
| Name | | Enter name |
| Telephone Number | | Enter telephone number |
| Email Address | | Enter email address |
| 5.* Are all participating NHS / HSC organisations undertaking the same protocol activities? | | |
| Yes | | |
| If ‘No’ give details of the activities taking place at NHS / HSC organisations that you will use this outline Organisation Information Document with. Additional outline Organisation Information Documents may be required for NHS / HSC organisations undertaking different activities. | | |
| If no, give details | | |

## Participating NHS / HSC Organisation Information

| **6. Name of Participating NHS / HSC Organisation**. If this Organisation Information Document is being used as an Agreement the name must be entered prior to agreement. |
| --- |
| **GP practices in England** |
| 7. Location/s: Please provide detail below where it is planned to undertake the research only at specified locations with the participating NHS / HSC organisation (i.e. hospital(s), GP Practice(s) and/or Research Unit(s)). It is not intended that the level of detail provided here captures individual departments within the participating NHS / HSC organisation. |

| Location (enter text below) | Activity (enter text below) |
| --- | --- |
| GP Practice | 1. Submit a practice profile including list size and federation status to assess eligibility. 2. Enter into a service level agreement (SLA) with Optimum Patient Care (OPC). Under the SLA OPC will provide a bespoke audit and feedback service to practices based on routine data extractions.   Practices will be randomized into control or IMP^2^ART practices**.**  IMP^2^ART practices will undertake the following activities:   - The whole practice team will attend a facilitation meeting with an educational facilitator (at the practice) to agree a plan for implementing the strategy IMP^2^ART (1hr) - The whole practice team will undertake an online introductory educational module which aims to raise the awareness of supported self-management (15-20 mins) - Key practice staff will undertake an in-depth supported self-management educational module (1 hr) - The practice will receive monthly audit and feedback reports from OPC allowing them to identify and target ‘at-risk’ patients for review.  \| - Practices will be provided with (‘QOF complaint) review templates that will be embedded in their system (EMIS, SystmOne, Vision, Microtest) for use in asthma reviews.  \| - Nurse specialists (employed by EfH; trained for IMP2ART) with experience of facilitation will facilitate implementation of IMP2ART within practices. \| \| --- \|   Control practices will continue to provide their usual asthma service.  Questionnaire Mailing  A random sample of practices (from both control and IMP^2^ART practices) will be selected to mail questionnaires. GPs will be asked to check patient lists for eligibility. The questionnaires will be mailed out by OPC on behalf of the practices.  Case Study   \| - Some practices will be invited to take part in an optional process evaluation to help the researchers gain some useful insights about self-management in primary care and experiences of taking part in the IMP^2^ART trial. If the practice decides to take part researchers would visit the practice to: - Observe anonymised practice documents and activities related to self-management (e.g. anonymised action plans, relevant local protocols, meetings or learning events) - Audio-record up to three asthma clinics; and - Interview individual staff members. \| \| --- \| \| \| --- \| --- \| --- \| |
|  |  |
|  |  |

| **8*. What is the role of the person responsible for research activities at the participating NHS / HSC organisation?**   - Principal Investigators are expected to be in place at participating NHS / HSC organisations where locally employed staff take responsibility for research procedures. In this scenario Principal Investigator should be selected even for single centre studies where the Chief Investigator will also be the Principal Investigator. - Where this is not the case, local collaborators are expected to be in place where central study staff will be present at the participating organisation to undertake research procedures (the role of the Local Collaborator is to facilitate the presence of Sponsor / CRO research staff). - Where existing data is being provided for research purposes without additional research procedures and without the presence of central research team members at the participating NHS / HSC organisation, select Chief Investigator. | |
| --- | --- |
| Local Collaborator | |
| **9. Contact** **details of person responsible for research activities at this participating NHS / HSC organisation as indicated in question 8 (if known).** If known, please enter the details of the person you have spoken to about their role in this study at this participating NHS / HSC organisation. If unknown, please leave blank and that person can be identified and listed here during the setup of the study. | |
| Name | Enter name |
| Post / Job Title | Enter post |
| Name of Employing Organisation | Enter name of participating NHS / HSC Organisation |
| Email Address | Enter email address |
| Telephone number | Enter telephone number |

## Timescales

| 10. Predicted Start and End Dates of the Study at this Participating NHS / HSC Organisation  The Sponsor or authorised delegate should propose a date on which it intends to start and complete research activity at this participating NHS / HSC organisation. Alternatively, this may be left blank when the Local Information Pack is shared, for agreement during study set up at the Participating NHS / HSC Organisation. | |
| --- | --- |
| Predicted Start Date (activities at this organisation) | 01/01/2020 |
| Predicted End Date (activities at this organisation) | 31/03/2024 |
| For many types of study the following dates are not applicable and this may be stated in answer. Where they are applicable, they should be provided by the Sponsor or authorised delegate before sharing the Local Information Pack, as indicative targets for agreement, or they may be negotiated between Sponsor or authorised delegate and participating NHS / HSC organisation after sharing the pack. | |
| Predicted Site Initiation Visit Date | 31/01/2020 |
| Predicted Start Date for participant recruitment | 31/01/2020 |
| Predicted End Date for participants recruitment (i.e. when the study moves into “follow up” activities.) | 31/01/2022 |
| Predicted End Date for all study activities  (i.e. “last patient visit” completed and study is ready to be archived.) | 31/03/2024 |

## Participant Numbers

| 11. How many research participants are expected at this participating NHS / HSC organisation?  For studies not directly involving human participants, please indicate the number of samples or data-sets to be obtained.  Please state if number of participants is per month, per year, overall, etc. |
| --- |
| 48 practices in Scotland; 96 practices in England |

## Study set up and delivery arrangements at Participating NHS / HSC Organisations

| 12*. The following are needed at the participating NHS / HSC organisation to deliver the study: e.g. specific equipment, patient/participant groups, service support, nursing time, etc*.* Please detail any specific requirements for participating NHS / HSC organisations to deliver this study, including by clarifying any requirements on participating NHS / HSC organisations relating to monitoring / self-monitoring, e.g. requirements for staff signature and delegation logs to be returned to the Sponsor and/or any particular access requirements that the Sponsor may have that it wishes to bring to the attention of the participating NHS / HSC organisation, likelihood of staff not employed at the participating NHS / HSC organisation coming on site, etc. |
| --- |
| Control Practices: Time for research lead to attend an initial researcher visit; time to allow OPC to set up data extraction.  IMP^2^ART practices: Time for research lead to attend an initial researcher visit; time to allow OPC to set up data extraction and install the asthma review template on the practice computer; time for whole practice team to attend an initial facilitation visit; time for all staff to complete module 1 of the online educational package; time of key staff to complete module 2 of the online educational package; time to review monthly audit and feedback reports.  Practices randomly selected to mail questionnaires: Time for the GP to check patient lists for eligibility.  Case Study Practices: Time to take part in process evaluation activities. |
| 13*. The following training will be provided by the Sponsor or authorised delegate for local research team members. Where only specific team members (e.g. the Principal Investigator) will receive this training, this should be specified. |
| IMP^2^ART practices: Staff will be provided with access to both online educational modules. Nurse specialists with experience of facilitation will facilitate implementation of IMP^2^ART within practices. |
| 14*. The Sponsor expects that local research team members will have the following skills and where they do not have those skills that they will undertake the relevant training before undertaking the relevant study activities*.* It would not be usual for the Sponsor to expect study specific training additional to that which it will provide. This section does however allow Sponsors to state, for example, that when they expect [training in Good Clinical Practice](https://www.hra.nhs.uk/about-us/news-updates/updated-guidance-good-clinical-practice-gcp-training/) for appropriate team members where the study is a Clinical Trial of an Investigational Medicinal Product, they will accept UK nationally recognised GCP training, training recognised on the [Transcelerate mutual recognition scheme](https://www.transceleratebiopharmainc.com/gcp-training-attestation/), etc. |
| No specific skills are required as the practices are participants and there is no requirement for local research team members. |
| 15*. The following funding/resources/equipment, etc. is to be provided to this participating NHS / HSC organisation. The Sponsor should answer this question whether this Organisation Information Document is to be used as the Agreement with the participating NHS / HSC organisation or not. Where the document is intended as the Agreement, further detail should be provided in Appendix 2. |
| \| Study set up – one off payment £40  Data extraction from OPC per time point (maximum of 3 time points) £23.21  Patient questionnaire check per time point (maximum 3 time points) £33  Interviews (GP) per hour £80  Interviews (Practice Manager, practice nurse) per hour £23.21  Case Study set up £300.00 \| \| --- \| |
|  |

##

## Appendices

## (Contents)

Appendix 1: General Provisions

Appendix 2: Finance Provisions

Appendix 3: Material Transfer Provisions

Appendix 4: Data Processing Agreement

Appendix 5: Data Sharing Agreement

Appendix 6: Intellectual Property Rights

**The sponsor or authorised delegate should answer the question at the top of Appendix 1 and, if it intends that this Organisation Information Document will be incorporated into an exchange of correspondence to form the Agreement (“Agreement”) between itself and the participating NHS / HSC organisation, the questions that appear at the top of each subsequent appendix.**

# Appendix 1: General Provisions

| ***Does the Sponsor intend that this Organisation Information Document forms the Agreement between itself and the participating NHS / HSC Organisation, or has a separate site agreement been provided?** | Organisation Information Document |
| --- | --- |
| It is recommended that the Organisation Information Document is used as the Agreement between Sponsor and participating NHS / HSC organisation for studies that are not clinical trials or investigations. The model Non-Commercial Agreement (mNCA) should be used for clinical trials or investigations.  Where the Organisation Information Document is to be used as the Agreement between the Sponsor and participating NHS organisation (hereafter singly “Party” or collectively the “Parties”), this document forms a formal legal contract between the Parties. In all cases where this document is the Agreement between the Parties, this Appendix 1 applies in full.  Additionally, the Sponsor or authorised delegate should use the questions at the top of each subsequent appendix to indicate whether or not that appendix also forms part of the Agreement.  Text highlighted in yellow is optional, including where alternative versions of the same clause may be used. The applicable option/s should be selected and text not to be used should be deleted prior to IRAS submission. No changes should be made to any text that does not appear in yellow highlight. | |

1. **OBLIGATIONS OF THE PARTIES**
   1. The Parties agree to comply with all relevant laws, regulations and codes of practice applicable to this Agreement including to the performance of the study. The Parties agree to comply with the World Medical Association Declaration of Helsinki, titled “Ethical Principles for Medical Research Involving Human Subjects” (where applicable) and the UK Policy Framework for Health and Social Care Research. The Parties shall conduct the study in accordance with:
      1. the Protocol, including appropriately made amendments thereto (which is/are hereby incorporated into this Agreement by reference);
      2. the terms of all relevant permissions and approvals. These may include, but are not limited to the terms and conditions of the favourable opinion given by the relevant NHS Research Ethics Committee, where applicable.
   2. The Parties shall carry out their respective responsibilities in accordance with this Agreement.
   3. The Parties agree to comply with all applicable statutory requirements and mandatory codes of practice in respect of confidentiality (including medical confidentiality) in relation to participants and study personnel.
   4. The Sponsor shall, on the giving of reasonable prior written notice to the Participating NHS / HSC Organisation, have the right to audit the Participating NHS / HSC Organisation’s compliance with this Agreement. The Sponsor may appoint an auditor to carry out such an audit. Such right to audit shall include access, during normal working hours to the Participating NHS / HSC Organisation's premises and to all relevant documents and other information relating to the study.
   5. The Participating NHS / HSC Organisation shall;
      1. promptly notify the Sponsor should any responsible body conduct or give notice of intent to conduct any inspection at the Participating NHS / HSC Organisation in relation to the study;
      2. allow the Sponsor to support the preparations for such inspection; and
      3. following the inspection, provide the Sponsor with the results of the inspection relevant to the study. The Sponsor will be responsible for sharing such results with the funder if required.
   6. In accordance with participant consent, the Participating NHS / HSC Organisation shall permit the Sponsor’s appointed representatives and any appropriately appointed monitor access to all relevant data for monitoring and source data verification. The Parties agree that such access will be arranged at mutually convenient times and on reasonable notice. Such monitoring may take such form as the Sponsor reasonably thinks appropriate including the right to inspect any facility being used for the conduct of the study, reasonable access to relevant members of staff at the Participating NHS / HSC Organisation and the right to examine any procedures or records relating to the study, subject at all times to clause 6 of this appendix. The Sponsor will alert the Participating NHS / HSC Organisation promptly to significant issues (in the opinion of the Sponsor) relating to the conduct of the study.
2. **LIABILITIES AND INDEMNITY**
   1. Nothing in this clause 2 shall operate so as to restrict or exclude the liability of a Party in relation to statutory or regulatory liability (including but not limited to breach of the data protection legislation), death or personal injury caused by the negligence or wilful misconduct of that Party or its agent(s), fraud or fraudulent misrepresentation or to restrict or exclude any other liability of a Party which cannot be so restricted or excluded in law.
   2. Where a Party is a non-NHS/HSC organisation, or an NHS/HSC organisation that is not a member of an NHS indemnity scheme, then that Party shall maintain all proper insurance or equivalent indemnity arrangements to cover liabilities arising from its participation in the study, in respect of any claims brought by or on behalf of a participant. Where the Party is an NHS/HSC organisation and is a member of an NHS indemnity scheme, it shall maintain its membership therein or otherwise ensure it has appropriate cover against claims arising as a result of clinical negligence by the Party and/or its agents brought by or on behalf of the participants. Each Party shall provide to the other such evidence of their insurance or equivalent indemnity cover maintained pursuant to clause 2.2 as the other Party shall from time to time reasonably request, such evidence might comprise confirmation that an NHS/HSC organisation is a member of one of the NHS indemnity schemes.
   3. Subject to clauses 2.4, 2.5, 2.6, 2.7 and 2.8, the legal entities that comprise the Sponsor, as specified under question 3 hereof (“Co-Sponsor institutions”), shall each indemnify the Participating NHS / HSC Organisation and its agents against any reasonable claims, proceedings and related costs, expenses, losses, damages and demands (“Claims”) in accordance with the applicable Co-Sponsor institution’s responsibilities and to the extent that the Claims arise or result from the respective negligent acts of, or omissions or wilful misconduct of a Co-Sponsor Institution, and/ or contracted third party, in its respective performance of this Agreement or in connection with the study.  For the avoidance of doubt, the Co-Sponsor institutions’ liability is not joint and several.
   4. Subject to clauses 2.3, 2.5, 2.6 and 2.8, the Participating NHS / HSC Organisation shall indemnify the each of the co-Sponsors and their respective agents, against any reasonable claims, proceedings and related costs, expenses, losses, damages and demands to the extent they arise or result from the negligent acts or omissions of, or the wilful misconduct of the Participating NHS / HSC Organisation, or its agents, in its performance of this Agreement or in connection with the study.
   5. An indemnity under clauses 2.3 or 2.4 shall only apply if the indemnified Party:
      1. informs the Party providing the indemnity in writing as soon as reasonably practicable following receipt of notice of the claim or proceedings;
      2. upon the indemnifying Party’s request and at the indemnifying Party’s cost gives the indemnifying Party full control of the claim or proceedings and provides all reasonable assistance; and
      3. makes no admission in respect of such claim or proceedings other than with the prior written consent of the indemnifying Party.
   6. Any indemnity under clauses 2.3 or 2.4 shall not apply to the extent any claims, proceedings and related costs, expenses, losses, damages or demands arise or result from the negligent acts or omissions or wilful misconduct or breach of statutory duty of the indemnified Party.
   7. The indemnity under clause 2.3 shall not apply to the extent any claims, proceedings and related costs, expenses, losses, damages or demands arise or result from:
      1. Participating NHS / HSC Organisation carrying out a treatment or procedure that would be routinely undertaken at or for that Participating NHS / HSC Organisation as part of National Health Service treatment; or
      2. Participating NHS / HSC Organisation preparing, manufacturing or assembling any equipment which is not done in accordance
         1. with the protocol; or
         2. with written instructions of the manufacturer; or
         3. (where such instructions differ from the instructions of the manufacturer) other written instructions of the Sponsor.
   8. No Party shall be liable to another in contract, tort/delict, breach of statutory duty or otherwise for any loss of profits, revenue, reputation, business opportunity, contracts, or any indirect, consequential or economic loss arising directly or indirectly out of or in connection with this Agreement.
   9. If a Party incurs any loss or damage (including costs and expenses) (“Loss”) arising or resulting from this Agreement and:
      1. All Parties are NHS bodies as defined in Section 9(4) of the National Health Service Act 2006 or Section 17 of the National Health Service (Scotland) Act 1978 or Section 7 (4) of the NHS (Wales) Act 2006 or Articles 16 and 26 of the Health and Personal Social Services (Northern Ireland) Order 1972, which established the Boards and Central Services Agency respectively and Article 10 of the Health and Personal Social Services (Northern Ireland) Order 1991: which established Trusts in Northern Ireland as appropriate; or
      2. One or more Party is a NHS body and the other Party (ies) is a NHS Foundation Trust; or
      3. All Parties are NHS Foundation Trusts;

Then clauses 2.10, 2.11 and 2.12 shall apply.

- 1. If all Parties are NHS bodies / NHS Foundation Trusts in England, Wales or Northern Ireland and are indemnified by the same indemnity scheme (being one of the NHS Resolution’s clinical negligence schemes or the Welsh Risk Pool or the Clinical Negligence Fund in Northern Ireland) and the Party incurring any loss can recover such loss under one of the indemnity schemes, then such Party shall rely on the cover provided by the indemnity scheme and not seek to recover the Loss from the other Party (ies). Where the other Party (ies) caused or contributed to the Loss, it undertakes to notify the relevant indemnity scheme(s) to take this into account in determining the future levies of all Parties in respect of the indemnity schemes.
  2. If:
     1. The Parties are members of the same indemnity scheme in England, Wales or Northern Ireland and the Party incurring the Loss is not indemnified for that Loss by its indemnity schemes; or
     2. All Parties are NHS bodies in Scotland; or
     3. The Parties are NHS bodies/Foundation Trusts established in different jurisdictions within the United Kingdom;

Then the Parties shall apportion such Loss between themselves according to their respective responsibility for such Loss.

- 1. If one or more Parties are NHS Foundation Trusts and the Party incurring the Loss is not responsible for all or part of the Loss and is not indemnified in respect of the Loss by one of the indemnity schemes then the Party incurring the Loss shall be entitled to recover the Loss from the other Party (ies) pursuant to the provisions of this Agreement.
  2. Subject to clause 2.1 and 2.7 the liability of the Participating NHS / HSC Organisation to the co-Sponsors and the liability of each of the co-Sponsors to the Participating NHS / HSC Organisation arising out of or in connection with any breach of this Agreement or any act or omission of either Party in connection with the performance of the study should be the greater of the amount of fees payable by the co-Sponsors to the Participating NHS / HSC Organisation under this Agreement or one hundred thousand (£100,000 GBP) pounds in respect of all, not each, co-Sponsors. For the avoidance of doubt, this cap applies also but not exclusively to the indemnities offered under clauses 2.3 and 2.4.
  3. Notwithstanding clause 2.13, in the case of equipment loaned by or on behalf of the Sponsor to the Participating NHS / HSC Organisation for the purposes of the study, the Participating NHS / HSC Organisation’s liability for damage to or loss of that equipment arising from its negligence shall exclude fair wear and tear and shall not exceed the replacement value of the equipment.

1. **PUBLICITY**
   1. None of the Parties shall use the name, logo or registered image of the other Parties or the employees of such other Party in any publicity, advertising or press release without the prior written approval of an authorised representative of that Party.
   2. The content and timing of any publicity, advertising or press release shall be agreed by all Parties, such agreement not to be unreasonably withheld.
2. **PUBLICATION**
   1. In accordance with all relevant laws, regulations and codes of practice, it is agreed that the Sponsor has an obligation to and shall publish the results of the full study and that the Participating NHS / HSC Organisation shall not publish any study data, including through presentation or submission of an abstract, without the prior permission in writing from the Sponsor (which shall not be unreasonably withheld or delayed).
3. **FREEDOM OF INFORMATION**
   1. Parties to this Agreement which are subject to the Environmental Information Regulations 2004 (EIR) and the Freedom of Information Act 2000 (FOIA) or the Freedom of Information (Scotland) Act 2002 (FOI(S)A) and which receive a request under EIR, FOIA or FOI(S)A to disclose any information that belongs to another Party shall notify and consult that Party, as soon as reasonably practicable, and in any event, not later than seven (7) working days after receiving the request.
   2. The Parties acknowledge and agree that the decision on whether any exemption applies to a request for disclosure of recorded information under EIR, FOIA or FOI(S)A is a decision solely for the Party responding to the request.
   3. Where the Party responding to an EIR, FOIA or FOI(S)A request determines that it will disclose information it will notify the other Party in writing, giving at least four (4) working days’ notice of its intended disclosure.
4. **CONFIDENTIALITY**
   1. Subject to clause 5 above, the Participating NHS / HSC Organisation agrees to treat the results, excluding any clinical data of the study, as confidential information of the Sponsor and the Sponsor agrees to treat personal data and confidential patient information as confidential information.
   2. The receiving Party agrees:
      1. To take all reasonable steps to protect the confidentiality of the confidential information and to prevent it from being disclosed otherwise than in accordance with this Agreement
      2. To ensure that any of its employees, students, researchers, consultants or sub-contractors who participate in the operation of the Study are made aware of, and abide by, the requirement of this clause 6.2.
      3. To use confidential information solely in connection with the operation of the Agreement and not otherwise, except in the case where the confidential information is personal data and/or confidential patient information, where it may be used solely on the basis of maintaining the common law duty of confidentiality and in accordance with the requirements of the data protection legislation, including but not limited to an appropriate legal basis/special category condition, appropriate transparency information and that the purpose is not incompatible with the original purpose.
      4. Not to disclose confidential information in whole or in part to any person without the disclosing Party’s prior written consent or, where the confidential information is personal data and/or confidential patient information, without maintaining the common law duty of confidentiality and in accordance with the requirements of the data protection legislation, including but not limited to an appropriate legal basis/special category condition, appropriate transparency information and that the purpose is not incompatible with the original purpose.
   3. The provision of clause 6.2 shall not apply to the whole or any part of the confidential information that is:
      1. lawfully obtained by the receiving Party free of any duty of confidentiality;
      2. already in the possession of the receiving Party and which the receiving Party can show from written records was already in its possession (other than as a result of a breach of clause 6.2.1 or 6.2.2);
      3. in the public domain (other than as a result of a breach of clause 7.2.1 or 7.2.2);
      4. independently discovered by employees of the receiving Party without access to or use of confidential information;
      5. necessarily disclosed by the receiving Party pursuant to a statutory obligation;
      6. disclosed with prior written consent of the disclosing Party;
      7. necessarily disclosed by the receiving Party by virtue of its status as a public authority in terms of the FOIA or the FOI(S)A;
      8. published in accordance with the provisions of clause 4.
   4. The restrictions contained in clauses 6.2 shall remain in force without limit in time in respect of personal data and any other information which relates to a patient, his or her treatment and/or medical records. Save as aforesaid and unless otherwise expressly set out in this Agreement, these clauses shall remain in force for a period of 10 years after the termination or expiry of this Agreement.

# Appendix 2: Finance Provisions

| Where this Organisation Information Document is to be used as the Agreement between Sponsor and Participating NHS / HSC organisation, please select one of the following. | |
| --- | --- |
| *****There are no funds/resources/equipment, etc. being provided to this participating NHS / HSC organisation by the Sponsor. This appendix should be left blank. | ☐ |
| *****This study involves the provision of funding/resource/equipment, etc. to the participating NHS / HSC organisation. This finance appendix forms part of the Agreement between the participating NHS / HSC organisation and the Sponsor. | ☑ |

### A. Financial Arrangements

The overall, study-wide recruitment for this study is competitive with a maximum figure of 144 Participants.  Once this target has been reached, the Sponsor will notify the Participating NHS / HSC Organisation.  No additional per participant payments will be made by the Sponsor to the Participating NHS / HSC Organisation for participants consented after such notification becomes effective.

|  | ***Area of Cost** | ***Payment (£ Sterling)** |
| --- | --- | --- |
| 1***** | Practice Liaison | £40.00 |
| 2***** | Data extraction | £23.21 per extraction |
| 3***** | Patient questionnaire check | 33.00 (three time points) |
| 4***** | Interviews (GP) | 80.00 per hour |
| 5***** | Interviews (Practice manager, practice nurse | 23.21 per hours |

If VAT is payable, then the Sponsor shall pay the VAT in addition to the payment of the agreed costs on presentation of a VAT invoice in which the VAT is stated as a separate item. Such invoices should quote the Participating NHS / HSC Organisation’s VAT registration number. If VAT is not payable, then the Sponsor shall issue a VAT exemption certificate.

Schedule of payments and details of payment arrangements

*****Invoices to be submitted quarterly to:

IMP^2^ART Study Programme Manager. Email:

*Usher Institute of Population Health Sciences and Informatics, University of Edinburgh, Doorway 3, Medical School, Teviot Place, Edinburgh EH8 9AG.*

**^**Payment to be made by cheque payable to:

[Insert NAME OF PARTICIPATING NHS / HSC ORGANISATION]

**^**and remitted to:

[Insert JOB TITLE/POSITION]

[Insert ADDRESS]

**^**Or arrange BACS Transfer to: [Insert BANK NAME].

**^**Sort code: [Insert SORT CODE]

**^**Account: [Insert ACCOUNT NUMBER]

**^**And send the relevant paper work to [Insert ADDRESSEE FOR PAPERWORK] at the above address

### B. Supplies Arrangements

Any equipment, materials, consumables, software or other items being provided by the Sponsor or procured by the participating organisation for use in the study shall be specified below.

Note 1: Parties should complete the table below. If the Participating NHS / HSC Organisation is to procure any items and is to be reimbursed by the Sponsor this should be specified in this appendix. Similarly if the Participating NHS / HSC Organisation is to pay the Sponsor for any items provided to the Participating NHS / HSC Organisation by or on behalf of the Sponsor this should be specified in this appendix.

Note 2: Parties should specify in this appendix, as appropriate, arrangements for:

- Ownership of items

- Insurance

- Storage instructions

- Instructions for use, return and/or destruction

- Any training to be provided

- Maintenance of equipment

| **Item** | **Quantity** | **Frequency of supply** | **Responsibility to supply/procure**  **(either Sponsor or Participating NHS / HSC Organisation only)** |
| --- | --- | --- | --- |
| Click here to enter text | Click here to enter text | Click here to enter text | Click here to enter text |
| Click here to enter text | Click here to enter text | Click here to enter text | Click here to enter text |
| Click here to enter text | Click here to enter text | Click here to enter text | Click here to enter text |
| Click here to enter text | Click here to enter text | Click here to enter text | Click here to enter text |
| Click here to enter text | Click here to enter text | Click here to enter text | Click here to enter text |

# Appendix 3: Material Transfer Provisions

| Where this Organisation Information Document is to be used as the Agreement between Sponsor and Participating NHS / HSC organisation, please select one of the following | |
| --- | --- |
| *****This study does not involve the transfer of human biological material (“Material”) from this participating NHS / HSC organisation to the Sponsor or its agents. This appendix does not form part of this Agreement. | ☑ |
| *****This study involves the transfer of human biological material from this participating NHS / HSC organisation to the Sponsor or its agents. These provisions form part of the Agreement between the Sponsor and this participating NHS / HSC organisation. | ☐ |

Material, as used in this appendix, means any clinical biological sample or portion thereof, derived from participants, including any information related to such Material, supplied by the Participating NHS / HSC Organisation to the either of the Co-Sponsors or their nominee.

1. In accordance with the protocol, the Participating NHS / HSC Organisation shall send Material to the a co-Sponsor or, in accordance with provision 7 below, to a third party nominated by the either of the co-Sponsors.
2. The Participating NHS / HSC Organisation warrants that all Material has been collected with appropriate informed consent and has been collected and handled in accordance with applicable law (including, without limitation, the Human Tissue Act 2004 or the Human Tissue (Scotland) Act 2006 (as the case may be)) and as required by the protocol.
3. Subject to provision 2 above, the Materials are supplied without any warranty, expressed or implied, including as to their properties, merchantable quality, fitness for any particular purpose, or that the Materials are free of extraneous or biologically active contaminants which may be present in the Materials.
4. One of the co-Sponsors shall ensure, or procure through an agreement with the co-Sponsor’s nominee as stated in provision 1 above that:
   1. the Material is used in accordance with the protocol, the consent of the participant, and the ethics approval for the study;
   2. the Material is handled and stored in accordance with applicable law;
   3. the Material shall not be redistributed or released to any person other than in accordance with the protocol or for the purpose of undertaking other studies approved by an appropriate ethics committee and in accordance with the participant’s consent.
5. The Parties shall comply with all relevant laws, regulations and codes of practice governing the research use of human biological material.
6. The Participating NHS / HSC Organisation and the a co-Sponsor shall each be responsible for keeping a record of the Material that has been transferred according to this appendix.
7. To the extent permitted by law the Participating NHS / HSC Organisation and its staff shall not be liable for any consequences of the supply to or the use by the co-Sponsor of the Material or of the supply to or the use by any third party to whom the co-Sponsor subsequently provides the Material or the co-Sponsor’s nominee as stated in provision 1 above, save to the extent that any liability which arises is a result of the negligence of the Participating NHS / HSC Organisation.
8. The co-Sponsor undertakes that, in the event that Material is provided to a third party in accordance with provision 2 above, it shall require that such third party shall undertake to handle any Material related to the study in accordance with all applicable statutory requirements and codes of practice and under terms no less onerous than those set out in this appendix.
9. Any surplus Material that is not returned to the Participating NHS / HSC Organisation or retained for future research (in line with participant consent) shall be destroyed in accordance with applicable law (including, without limitation, the Human Tissue Act 2004 or the Human Tissue (Scotland) Act 2006 (as the case may be)).

**These provisions do not remove the need for the Sponsor to clearly lay out in their protocol (and to potential participants in the participant information) at a minimum the following information for all Material taken: 1) The nature of the Materials, 2) The reason that the Material is being taken, 3) where the Material is to be sent and, 4) what will happen to any remaining Material once it has been processed/analysed, etc. for the purposes of this study (e.g. return, retention or destruction). Detailed guidance on what information should be included in a protocol may be found on the HRA website:* [*www.hra.nhs.uk*](http://www.hra.nhs.uk)

# Appendix 4: Data Processing Agreement

| Where this Organisation Information Document is to be used as the Agreement between Sponsor and Participating NHS / HSC organisation, please select one of the following. | |
| --- | --- |
| *****This study does not involve any processing of personal data by this participating NHS / HSC organisation on behalf of the Sponsor. This appendix does not form part of this Agreement. | ☑ |
| *****This study involves processing of personal data by this participating NHS / HSC organisation on behalf of the Sponsor. These provisions form part of the Agreement between the Sponsor and this participating NHS / HSC organisation.  For the avoidance of doubt, when used, these provisions are intended to form a legally binding contractual obligation for the purposes of compliance with the GDPR, specifically GDPR Article 28 (3). | ☐ |

1. For the purposes of the data protection legislation, the Sponsor is the controller and the Participating NHS / HSC Organisation is the Sponsor's processor in relation to all processing of personal data that is processed for the purpose of this study and for any future research use under the controllership of the Sponsor, that would not have taken place but for this Agreement regardless where that processing takes place.
2. The Parties acknowledge that whereas the Sponsor is the controller in accordance with Clause 1 of this appendix, the Participating NHS / HSC Organisation is the controller of the personal data collected for the purpose of providing clinical care to the participants. This personal data may be the same personal data, collected transparently and processed for research and for care purposes under the separate controllerships of the Sponsor and Participating NHS / HSC Organisation.
3. Where the Participating NHS / HSC Organisation is the Sponsor's processor and thus where the processing is undertaken by the Participating NHS / HSC Organisation for the purposes of the study, Clauses 5.a. to 5.j below will apply. For the avoidance of doubt, such Clauses do not apply where the Participating NHS / HSC Organisation is processing the participant personal data as a controller.
4. The Participating NHS / HSC Organisation agrees only to process personal data for and on behalf of the Sponsor in accordance with the instructions of the Sponsor and for the purpose of the study and to ensure the Sponsor’s compliance with the data protection legislation;
5. The Participating NHS / HSC Organisation agrees to comply with the obligations applicable to processors described by Article 28 GDPR including, but not limited to, the following:
   1. to implement and maintain appropriate technical and organisational security measures sufficient to comply at least with the obligations imposed on the controller by Article 28(1);
   2. to not engage another processor without the prior written authorisation of the Sponsor (Article 28(2)) ;
   3. to process the personal data only on documented instructions from the Sponsor unless required to do otherwise by legislation, in which case the Participating NHS / HSC Organisation shall notify the Sponsor before processing, or as soon as possible after processing if legislation requires that the processing occurs immediately, unless legislation prohibits such notification on important grounds of public interest (Article 28(3a)).;
   4. to ensure that personnel authorised to process personal data are under confidentiality obligations (Article 28(3b));
   5. to take all measures required by Article 32 GDPR in relation to the security of processing (Article 28(3c));
   6. to respect the conditions described in Article 28(2) and (4) for engaging another processor (Article 28(3d));
   7. to, taking into account the nature of the processing, assist the Sponsor, by appropriate technical and organisational measures, insofar as this is possible, to respond to requests for exercising data subjects’ rights (Article 28(3e));
   8. to assist the controller, to ensure compliance with the obligations pursuant to Articles 32 to 36 GDPR taking into account the nature of the processing and the information available to the Participating NHS / HSC Organisation (Article 28(3f));
   9. to, at the choice of the Sponsor, destroy or return all personal data to the Sponsor at the expiry or early termination of the Agreement, unless storage is legally required (Article 28(3g)) or where that personal data is held by the Participating NHS / HSC Organisation as controller for the purpose of clinical care or other legal purposes; and
   10. to maintain a record of processing activities as required by Article 30(2) GDPR.
6. The Participating NHS / HSC Organisation shall ensure that:
   1. its agents do not process personal data except in accordance with this Agreement (and in particular the protocol);
   2. it takes all reasonable steps to ensure the reliability and integrity of any of its agents who have access to the personal data and ensure they:
      1. are aware and comply with the Participating NHS / HSC Organisation 's duties under this clause;
      2. are subject to mandatory training in their information governance responsibilities and have appropriate contracts including sanctions, including for breach of confidence or misuse of data; and
      3. are informed of the confidential nature of the personal data and understand the responsibilities for information governance, including their obligation to process personal data securely and to only disseminate or disclose for lawful and appropriate purposes.
7. The Participating NHS / HSC Organisation agrees to:
   1. allow the Sponsor(s) or another auditor appointed by the Sponsor(s) to audit the Participating NHS / HSC Organisation’s compliance with the obligations described by this Appendix, data protection legislation in general and Article 28 GDPR in particular, on reasonable notice subject to the Sponsor complying with all relevant health and safety and security policies of the participating site and/or to provide the Sponsor with evidence of its compliance with the obligations set out in this Agreement; and
   2. obtain prior agreement of the Sponsor to store or process personal data outside the European Economic Area.
8. Where the Participating NHS / HSC Organisation stores or otherwise processes personal data outside of the European Economic Area as the Sponsor’s processor, it warrants that it does so in compliance with the Data Protection Legislation.

# Appendix 5: Data Sharing Agreement

| Where this Organisation Information Document is to be used as the Agreement between Sponsor and Participating NHS/HSC organisation, please select one of the following | |
| --- | --- |
| *****This study does not involve the transfer of personal data from this participating NHS / HSC organisation to the Sponsor or its agents, nor is there transfer of confidential information between the Parties. This appendix does not form part of this Agreement. | ☑ |
| *****This study involves the transfer of personal data from this participating NHS / HSC organisation to the Sponsor or its agents, and/or there is transfer of confidential information between the Parties. These provisions form part of the Agreement between the Sponsor and this participating NHS / HSC organisation. | ☐ |

1. Personal data shall not be disclosed to the Sponsor by the participating NHS / HSC organisation, save where this is required directly or indirectly to satisfy the requirements of the protocol, or for the purpose of monitoring or reporting adverse events, or in relation to a claim or proceeding brought by a participant in connection with the study.
2. The Sponsor agrees to use personal data solely in connection with the operation of the Agreement, or otherwise for purposes not incompatible with this original purpose (Article 5, 1 (b) GDPR), and not otherwise. In particular,
   1. Not to disclose personal data to any person except in accordance with applicable legal requirements and codes of practice.
3. The Sponsor agrees to comply with the obligations placed on a controller by the data protection legislation. This is not limited to, but includes, being responsible for and able to demonstrate compliance with the principles relating to processing of personal data (Article 5 GDPR)
4. The Sponsor agrees to ensure persons processing personal data under this Agreement are equipped to do so respectfully and safely. In particular:
   1. To ensure any persons (excluding employees, honorary employees, students, researchers, consultants and subcontractors of the participating NHS / HSC organisation) processing personal data understand the responsibilities for information governance, including their obligation to process personal data securely and to only disseminate or disclose for lawful and appropriate purposes.
   2. To ensure any persons (excluding employees, honorary employees, students, researchers, consultants and subcontractors of the Participating NHS / HSC Organisation) have appropriate contracts providing for personal accountability and sanctions for breach of confidence or misuse of data including deliberate or avoidable data breaches.
5. The Sponsor agrees to proactively prevent data security breaches and to respond appropriately to incidents or near misses. In particular,
   1. To ensure that personal data are only accessible to persons who need it for the purposes of the study and to remove access as soon as reasonably possible once it is no longer needed.
   2. To ensure all access to personal data on IT systems processed for study purposes can be attributed to individuals.
   3. To identify, review and improve processes which have caused breaches or near misses, or which force persons processing personal data to use workarounds which compromise data security.
   4. To adopt measures to identify and resist cyber-attacks against services and to respond to relevant external security advice.
   5. To take action immediately following a data breach or near miss.
6. The Sponsor agrees to ensure personal data are processed using secure and up to date technology. In particular,
   1. To ensure no unsupported operating systems, software or internet browsers are used to support the processing of personal data for the purposes of the study.
   2. To put in place a strategy for protecting relevant IT systems from cyber threats which is based on a proven cyber security framework such as Cyber Essentials.
   3. To ensure IT suppliers are held accountable via contracts for protecting personal data they Process and for meetings all relevant information governance requirements.

# Appendix 6: Intellectual Property Rights

| Where this Organisation Information Document is to be used as the Agreement between Participating NHS / HSC organisation, please select one of the following* | |
| --- | --- |
| *****This study does not require the protection of background intellectual property rights, nor is there potential for the generation of new intellectual property. This appendix does not form part of this Agreement. | ☑ |
| *****This study requires the protection of background intellectual property rights, and / or there is potential for the generation of new intellectual property. These provisions form part of the Agreement between the Sponsor and this participating NHS / HSC organisation. | ☐ |

1. All background intellectual property rights (including licences) and know how and their improvements used in connection with the Study shall remain the property of the Party introducing the same and the exercise of such rights for purposes of the Study shall not knowingly infringe any third party’s rights.
2. All intellectual property rights and know how in the Protocol, and in the study data, excluding clinical procedures developed or used by the Participating NHS / HSC Organisation independently of the Study, shall belong to the Sponsor.  The Participating NHS / HSC Organisation hereby assigns all such intellectual property rights, and undertakes to disclose all such know how, to the Sponsor.
3. Subject to clause 1 and 2, all intellectual property rights deriving or arising from the Material or any derivations of the Material provided to the Sponsor by the Participating NHS / HSC Organisation shall belong to the Sponsor.
4. At any time within the duration of the Study, the Participating NHS / HSC Organisation shall at the request and expense of the Sponsor execute all such documents and do all acts necessary to fully vest the intellectual property rights in the Sponsor.  To give effect to this clause 4, the Participating NHS / HSC Organisation shall ensure that its agents involved in the Study assign such intellectual property rights falling within clauses 2 and 3 and disclose such know how to the Participating NHS / HSC Organisation.
5. Subject to this Clause 5 and Clause 6, nothing in this Appendix shall be construed so as to prevent or hinder the Participating NHS / HSC Organisation from using its own know how or clinical data gained during the performance of the Study, at its own risk, in the furtherance of its normal activities of providing clinical care to the extent that such use does not result in the disclosure or misuse of confidential information or the infringement of an intellectual property right of the Sponsor, or their funder.  This clause 5 does not permit the disclosure of any of the study data, all of which remain confidential until publication of the results. Any study data not so published remains the confidential information of the Sponsor, or their funder.
6. The Participating NHS / HSC Organisation may, with the prior written permission of the Sponsor (such permission not to be unreasonably withheld), use study data gained during the performance of the Study, at its own risk, in the furtherance of its normal activities of commissioning clinical services, teaching and research to the extent that such use does not result in the disclosure or misuse of confidential information or the infringement of an intellectual property right of the Sponsor or their funder.  This clause 6 does not permit the disclosure of any of the study data, all of which remain confidential until publication of the results of the Study.

**Authorisation When Using This Organisation Information Document as An Agreement**

**(when used as an Agreement, the Participating NHS Organisation is a “Party” to the Agreement and the Sponsor is a “Party” to the Agreement – collectively the “Parties”).**

| **Authorisation on behalf of Participating NHS / HSC Organisation**  It is not intended that this confirmation requires wet-ink signatures, or a passing of hard copies between the Sponsor and participating NHS / HSC organisation. Instead, Sponsors are expected to accept confirmation by email from an individual empowered by the Participating NHS / HSC Organisation to agree to the commencement of research (including any budgetary responsibility, where the study involves the transfer of funds). |
| --- |

| **^ The Participating NHS / HSC Organisation confirms (by checking the box) that the Principal Investigator, where one is required, is aware of and has agreed to discharge their responsibilities in line with the** [**UK Policy Framework for Research and Social Care**](https://www.hra.nhs.uk/planning-and-improving-research/policies-standards-legislation/uk-policy-framework-health-social-care-research/).**.** | ☑ |
| --- | --- |
| **^ The Participating NHS / HSC Organisation has considered and mitigated any conflict/s of interest declared by the principal investigator.** |  |
| If yes, please detail conflict of interest | |

| *** Authorised on behalf of Sponsor by:** | |
| --- | --- |
| **Name** |  |
| **Job Title** |  |
| **Organisation Name** |  |
| **Date** |  |
| **^ Authorised on behalf of Participating NHS / HSC Organisation by:** | |
| **Name** |  |
| **Job Title** |  |
| **Organisation Name** |  |
| **Date** |  |
